# Type IV collagen turnover is predictive of mortality in COPD: a comparison to fibrinogen in a prospective analysis of the ECLIPSE cohort

**DOI:** 10.1186/s12931-019-1026-x

**Published:** 2019-04-01

**Authors:** Sarah Rank Rønnow, Jannie Marie Bülow Sand, Lasse Løcke Langholm, Tina Manon-Jensen, Morten Asser Karsdal, Ruth Tal-Singer, Bruce E. Miller, Jørgen Vestbo, Diana Julie Leeming

**Affiliations:** 1 Nordic Bioscience A/S, Herlev, Denmark; 2 University of Southern Denmark, The Faculty of Health Science, Odense, Denmark; 3 The Faculty of Health and Medical sciences, University of Copenhagen, Copenhagen, Denmark; 4 GSK R&D, Collegeville, PA USA; 5 Division of Infection Immunity and Respiratory Medicine, The University of Manchester, Manchester Academic Health Science Centre, and Manchester University NHS Foundation Trust, Manchester, England

**Keywords:** COPD, Biomarkers, Basement membrane, Collagen type IV, ECLIPSE

## Abstract

**Background:**

Identifying subjects with chronic obstructive pulmonary disease (COPD) at high risk of exacerbation and mortality is key to aid individual management of COPD. The only FDA approved blood-based drug development biomarker for patients at high risk of mortality, is plasma fibrinogen. In this study, we benchmarked two biomarkers of basement membrane remodeling, a characteristic of COPD, against plasma fibrinogen alone and as a combination. The biomarkers of basement membrane remodeling are two neoepitopes from of the alpha 3 chain of type IV collagen (COL4A3).

**Materials and methods:**

COL4A3 degradation was assessed by the biomarkers C4Ma3 and tumstatin (TUM) in year 1 plasma samples in 984 COPD subjects, 95 non-smoking controls and 95 smoking controls from the Evaluation of COPD Longitudinally to Identify Predictive Surrogate End-points (ECLIPSE) cohort. They were measured by competitive ELISA using monoclonal antibodies recognizing two specific MMP-generated cleavage site within COL4A3. The level of fibrinogen was previously assessed in year 1 plasma.

**Results:**

In COPD subjects, plasma C4Ma3 levels were significantly correlated with plasma fibrinogen levels (0.389 (*P* < 0.0001)). Cox proportional-hazards regression adjusted for relevant confounders showed that high levels of plasma C4Ma3, but not TUM, were related to a higher risk of mortality (hazard ratio 5.12 (95% CI 2.28–11.50), *P* < 0.0001). High levels of plasma fibrinogen were not associated with all-cause mortality in this subpopulation, contradictory to published results. Whereas plasma C4Ma3 multiplied by fibrinogen showed to be related to a higher risk of mortality (hazard ratio 5.74 (95% CI 2.65–12.41), *P* < 0.0001). Plasma C4Ma3 levels were related to the number of hospitalizations due to COPD exacerbations in the year before study start (*P* = 0.0375). Fibrinogen levels were related to hospitalized exacerbations prior to study start (*P* = 0.0058) and were also related to future exacerbations (*P* < 0.0001).

**Conclusion:**

We compared herein fibrinogen, C4Ma3 and TUM as biomarkers for COPD prognosis. Fibrinogen was related to future exacerbation, whereas C4Ma3 and the combination of C4Ma3 with fibrinogen were superior to fibrinogen alone in predicting mortality. This pilot study suggests that the assessment of plasma C4Ma3 could be important for identifying COPD patients with a poor prognosis.

**Trial registration:**

NCT00292552, GSK Study No. SCO104960.

**Electronic supplementary material:**

The online version of this article (10.1186/s12931-019-1026-x) contains supplementary material, which is available to authorized users.

## Background

Chronic obstructive pulmonary disease (COPD) is the third leading cause of death worldwide [1]. It is characterized by progressive airflow limitation related to chronic inflammation, lung tissue destruction and small airway fibrosis. Identifying patients at risk of progression as defined by a rapid decline in forced expiratory volume in one second (FEV_1_), an increased rate of exacerbations or mortality is challenging but crucial for precision medicine. Plasma fibrinogen was the first blood-based biomarker of COPD to be qualified approved by the U.S. Food and Drug Administration (FDA) in 2015 for the enrichment of subjects with COPD at higher risk of progression in clinical trials [2, 3]. The approval was based on data from the ECLIPSE cohort, showing its value as a prognostic biomarker to identify COPD subjects at risk of exacerbations or all-cause mortality.

One of the main structural characteristics of COPD is changes to the lung tissue due to altered extracellular matrix (ECM) content and remodeling of the ECM. In the distal airways and alveoli, the ECM is mainly represented by the basement membrane (BM) with only a thin layer of or no interstitial matrix. The ECM layer is thin, thus ensuring easy transport of gases between the air and the blood [4–6], and the alveoli contain a specialized BM that is optimal for this gas transport. There are six distinct type IV collagen α-chains, namely α1(IV) through α6(IV), which are selectively expressed in different membranes at various stages of development and in specific tissues [5, 7]. The α1α1α2(IV) isoform is ubiquitous, but the alveolar BM, as well as the glomerular BM, also contains the specialized α3α4α5(IV) isoform that facilitates gas exchange [8, 9]. The function of type IV collagen in both lungs and kidneys indicates that this specialized isoform is important for the diffusion across the BM barrier in these two organs. The α3α4α5(IV) isoform has been shown to contain a high number of disulfide cross-links and consequently to be more resistant to proteolytic degradation than α1α1α2(IV). Thus, the α3α4α5(IV) may be better suited for the more vulnerable position in the alveolar BM [10]. This makes α3α4α5(IV) a very specific collagen type IV isoform of the lungs.

Alterations in the BM influence the processes of lung injury and repair, implying an important role for type IV collagen in pulmonary diseases. For example, the disruption of the alveolar BM as a result of matrix metalloproteinase (MMP) activity is a consistent finding in idiopathic pulmonary fibrosis (IPF) [11], that may contribute to aberrant lung remodeling by hindering re-epithelialization and enhancing fibroblast invasion mediated by increasing permeability [12]. Thus, the remodeling of type IV collagen may also be crucial in the pathogenesis of COPD.

Fibrinogen is a coagulation factor that is essential for the blood clotting process in tissues following injury. Plasma levels of fibrinogen have been shown to predict acute exacerbations of COPD as well as all-cause mortality [2, 3]. However, plasma fibrinogen levels may rise in response to extrapulmonary injury and systemic inflammation in COPD which makes it less specific for disease activity specifically in the lung.

In this study, we investigated BM remodeling as a risk biomarker for COPD disease progression as defined by alterations in lung tissue structure. More specifically, we evaluated the alveolar BM remodeling by measuring the serological biomarker (C4Ma3) reflecting MMP-mediated degradation of the type IV collagen alpha 3 chain and tumstatin (TUM) the C-terminal non-collagenous (NC1) domain of the type IV collagen alpha 3 chain. BM remodeling may be more specific for the changes that occur in the lung of COPD patients as compared to plasma fibrinogen and therefore potentially a better prognostic biomarker alone or incremental to the prognostic value of fibrinogen. To investigate this, we benchmarked the performance of BM remodeling against plasma fibrinogen and a combination of plasma C4Ma3 and fibrinogen in relation to acute exacerbations and mortality outcome in subjects with COPD. We conducted a retroprospective analysis of the plasma C4Ma3, TUM and fibrinogen in a subset of the three-year Evaluation of COPD Longitudinally to Identify Predictive Surrogate End-points (ECLIPSE) study.

## Methods

### Study design

The study design of ECLIPSE (clinicaltrials.gov identifier NCT00292552; GSK study code SCO104960) has been described in detail previously [13, 14]. Briefly, ECLIPSE is an observational, longitudinal study which evaluated participants at baseline and at months three, six, and subsequently every six months for three years. For the current analyses, we used clinical and biomarker data obtained at year one and year three. Death was recorded up to day 1060 in year three of the study. Cause of death was not available for the present analyses. ECLIPSE complies with the Declaration of Helsinki and Good Clinical Practice Guidelines and has been approved by the ethics committees of the participating centers. All participants provided written informed consent before the performance of any study-related assessments.

### Population

**Fig. 1:**
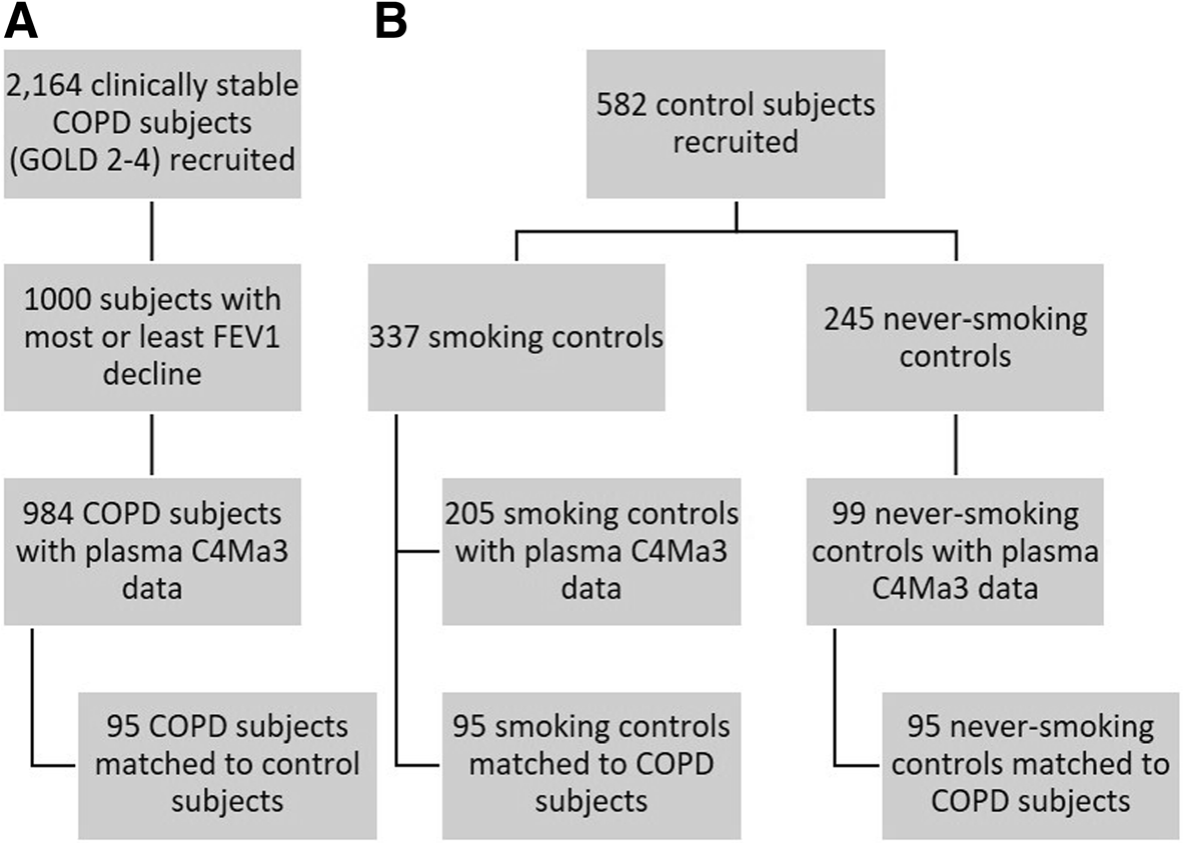
Study recruitment and participant flow. **a** COPD participants. **b** controls

The full ECLIPSE study included 2164 participants diagnosed with COPD based on a post-bronchodilator FEV_1_ < 80% of the reference value, FEV_1_/forced vital capacity (FVC) ≤ 0.7, and a smoking history of ≥10 pack-years [13, 14]. The cohort was recruited from the outpatient clinics of the participating centers. The current study was performed on a subpopulation of 1000 COPD subjects of the ECLIPSE cohort composed of 500 subjects progressing the most and 500 subjects progressing the least as defined by FEV_1_ decline over the three-year study period. Individuals with available plasma C4Ma3 data were included in the analyses, giving a total of 984 COPD participants (Fig. 1a). Additionally, plasma C4Ma3 data for smoking (*n* = 95) and never-smoking (*n* = 95) controls age gender and body mass index (BMI) matched to 95 of the COPD subjects were included in the analyses (Fig. 1).

### Measurements of serological biomarkers

Plasma (heparin anticoagulant) samples were collected from participants in the fasting state at year 1 by venipuncture into vacutainer tubes followed by centrifugation at 2000 g for 10–15 min. Plasma was stored at -80 °C until analyzed. Plasma C4Ma3 and TUM levels were measured by validated competitive ELISAs utilizing monoclonal antibodies targeting a specific neo-epitope of type IV collagen generated by MMP cleavage of the alpha 3 chain (Nordic Bioscience, Herlev, Denmark) [15, 16]. Measurements were performed in a blinded manner according to the manufacturer’s instructions. Analytes detected by this assay are stable in plasma samples that have undergone at least four freeze/thaw cycles [15]. Prior to this study, plasma fibrinogen levels were measured in year one plasma (dry ethylenediaminetetraacetic acid anticoagulant) samples as described previously [17]. As measurements were performed in plasma and not in serum, in order to be able to compare fibrinogen results to the typically used liquid sodium citrate anti-coagulant, the published cut-off level, plasma fibrinogen levels were corrected by − 13.6%, as described in the statistical analysis plan from the COPD Biomarkers Qualification Consortium for their FDA application (Supplements for [18]).

### Statistical analysis

Basic demographics were compared using Mann-Whitney U test, Kruskal-Wallis test, or chi-squared test as appropriate. Plasma C4Ma3, TUM and fibrinogen levels were compared between groups or with clinical parameters using Spearman’s rank correlation, Mann-Whitney U test or Kruskal-Wallis test as appropriate.

The association of plasma C4Ma3 and plasma fibrinogen with future and prior acute exacerbations was investigated by logistic regression.

Cox proportional hazards analyses were used to assess the prognostic value of plasma C4Ma3, TUM and fibrinogen levels for all-cause mortality for one standard deviation (SD) increase in the biomarker. Plasma C4Ma3 data were dichotomized based on receiver operating curve (ROC) analyses. The ROC curve had an area under the curve (AUC) of 0.68 (95% CI 0.65–0.71), *P* = 0.0005, and gave a cut-off of 5.5 ng/mL for the identification of subjects who died with a sensitivity of 40.0 and specificity of 90.8. Plasma fibrinogen data were dichotomized based on the published cut-off level of 350 mg/dL. A cut-off value for plasma C4Ma3 multiplied by plasma fibrinogen (C4Ma3*Fibrinogen) was defined by a ROC analysis for identification of COPD subjects at risk of mortality. The ROC curve had an AUC of 0.68 (95% CI 0.58–0.80, *P* = 0.0004) and gave a cut-off of 2037 for the identification of subjects who died with a sensitivity of 53.3 and specificity of 83.9. Mortality risk for groups below and above the cut-off was compared using Cox proportional hazards analysis with or without relevant confounders as determined by univariate Cox proportional hazards analysis. Kaplan-Meier survival curves compared the mortality risk for patients belonging to the high vs. low biomarker groups. All tests performed (MedCalc Statistical Software version 16.8.4, MedCalc Software bvba, Ostend, Belgium) were two-sided at the 0.05 level of significance, and all *P* values are nominal as no adjustments were made for multiple comparisons.

## Results

### C4Ma3 is increased in COPD patients

**Fig. 2:**
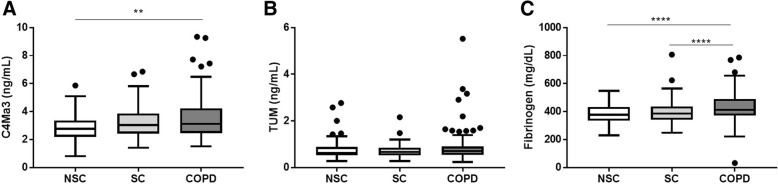
Plasma C4Ma3 (**a**), TUM (**b**) and fibrinogen (**c**) levels in matched never-smoking controls (NSC), smoking controls (SC), and COPD subjects. Each group *n* = 95. Data are shown as Tukey box-and-whiskers plot with a line indicating median, the box indicating the 25th and 75th percentiles (the interquartile range, IQR), and whiskers indicating 1.5 times the IQR. Data points outside this limit are shown by individual data points. Data were analyzed by Kruskal-Wallis test using Dunn’s multiple comparison. Asterisks indicate statistical significance: **p < 0.01, and ****p < 0.0001

Levels of plasma C4Ma3 were significantly different between COPD and matched control participants (*P* = 0.0047). Group analysis showed that plasma C4Ma3 levels were significantly elevated in COPD subjects (median 38 pack years) as compared to never-smoking, but not smoking controls (median 30 pack-years) (Table 1/Fig. 2). Plasma fibrinogen level was significantly increased in COPD subjects compared to smoking controls and never-smoking controls (Table 1/Fig. 2). Whereas, TUM did not show any difference between the three groups (Fig. 2). In COPD subjects, plasma C4Ma3 levels were significantly correlated with plasma fibrinogen levels (Spearman’ correlation coefficient 0.389 (*P* < 0.0001).

**Table 1:** Demographic characteristics of COPD subjects and controls

Parameter	Smoking controls	Never-smoking controls	COPD	*P* value
*n*	*95*	*95*	*95*	
Age, mean (SD)	59.4 (7.2)	58.8 (7.6)	60.0 (6.4)	0.663 ¥
Female gender, n (%)	47 (49%)	47 (49%)	47 (49%)	1.000 £
BMI, mean (SD)	27.4 (4.3)	28.4 (4.5)	27.4 (5.5)	0.239 ¥
Pack-years, median (IQR)	30 (18–40)	0 (0–0)	38 (30–48)	< 0.0001 ¥
Current smokers, n (%)	47 (49%)	0 (0%)	47 (49%)	< 0.0001 £
Cardiovascular history, n (%)	26 (27%)	16 (17%)	21 (22%)	0.217 £
Post bronchodilator FEV_1_ (% predicted), mean (SD)	110.3 (13.6)	114.0 (13.7)	53.3 (15.6)	< 0.0001 ¥
C4Ma3 (ng/mL), median (IQR)	3.04 (2.44–3.85)	2.76 (2.20–3.35)	3.12 (2.46–4.22)	0.0047 ¥
TUM (ng/mL), median (IQR)	0.71 (0.64–0.78)	0.63 (0.60–0.73)	0.73 (0.63–0.80)	0.263 ¥
Fibrinogen (mg/dL), median (IQR)	343.4 (311.0–383.6)	326.6 (287.7–375.8)	356.4 (318.8–421.2)	0.0009 ¥

¥ Kruskal-Wallis; £ Chi-squared test

### Basic demographics

Basic demographics for the full COPD population are shown in Table 2. Mean age was 63.1 (7.2) years, BMI was 26.8 (5.9), and 357 (36%) were female. Mean pack-years were 47.4 (26.0) and 363 (37%) were current smokers. Post-bronchodilator FEV_1_ was a mean of 50.6% (15.2) of predicted value, 558 (57%) had significant emphysema, 439 (45%) reported one or more exacerbation in the year prior to study start, and 30 (3%) died from year one to the end of the study period (year three).

**Table 2:** Basic Demographics

Parameter	COPD	Survivors	Deceased	*P* value (survivors vs. deceased)
n	984	954	30	
Age, mean (SD)	63.1 (7.2)	62.9 (7.2)	68.2 (6.3)	< 0.0001 ¥
Female gender, n (%)	357 (36%)	346 (36%)	11 (37%)	0.964 £
BMI, mean (SD)	26.8 (5.9)	26.8 (5.8)	27.3 (7.8)	0.777 ¥
Pack-years, mean (SD)	47.4 (26.0)	47.1 (25.3)	55.9 (42.5)	0.934 ¥
Current smokers, n (%)	363 (37%)	360 (38%)	3 (10%)	0.002 £
Cardiovascular history, n (%)	310 (32%)	298 (31%)	12 (40%)	0.309 £
Post bronchodilator FEV_1_ (% predicted), mean (SD)	50.6 (15.2)	50.6 (15.3)	48.5 (13.0)	0.384 ¥
GOLD stage, n (%)				0.695 £
2	487 (49%)	473 (50%)	14 (47%)	
3	397 (40%)	383 (40%)	14 (47%)	
4	100 (10%)	98 (10%)	2 (7%)	
GOLD stage, n (%)				0.231 £
A	250 (25%)	244 (26%)	6 (20%)	
B	128 (13%)	121 (13%)	7 (23%)	
C	226 (23%)	222 (23%)	4 (13%)	
D	339 (34%)	329 (34%)	10 (33%)	
mMRC score, median (IQR)	1 (1–2)	1 (1–2)	2 (1–3)	0.022 ¥
SGRQ-C total score, median (IQR)	47.2 (33.2–60.6)	47.2 (33.2–60.2)	53.3 (33.0–66.0)	0.294 ¥
6MWD (meters), mean (SD)	385.4 (119.0)	387.0 (119.1)	335.3 (105.0)	0.013 ¥
FV950 baseline, mean (SD)	16.3 (11.3)	16.2 (11.3)	17.4 (10.5)	0.425 ¥
Emphysema (%LAA ≥ 10%), n (%)	558 (57%)	538 (56%)	20 (67%)	0.264 £
Number of hospitalized exacerbations in year prior to study, mean (SD)	0.16 (0.53)	0.16 (0.52)	0.37 (0.85)	0.036 ¥
BODE index, median (IQR)	3.0 (1.0–4.0)	3.0 (1.0–4.0)	3.5 (2–5)	0.065 ¥
Dead by end of study, n (%)	30 (3%)	0 (0%)	30 (100%)	< 0.0001 £
Survival time (days), mean (SD)	1056 (28)	1060 (0)	922 (84)	< 0.0001 ¥
C4Ma3 (ng/mL), median (IQR)	3.28 (2.55–4.33)	3.25 (2.54–4.30)	4.02 (3.16–6.05)	0.0008 ¥
TUM (ng/mL), median (IQR)	0.71 (0.69–0.74)	0.71 (0.69–0.73)	0.79 (0.71–1.17)	0.0467 ¥
Fibrinogen (mg/dL), median (IQR)	378.4 (334.4–434.1)	377. 6 (333.5–432.0)	445.4 (359.4–527.0)	0.0006 ¥

¥ Mann-Whitney; £ Chi-squared test. Data are shown as mean ± SD, median (25th, 75th) or number (%). *BMI* body mass index, *FEV*_*1*_ post-bronchodilator forced expiratory volume in 1 s, *GOLD* global initiative for chronic obstructive lung disease, *mMRC* modified Medical Research Council dyspnea score, *SGRQ* St. George’s respiratory questionnaire, *6MWD* 6-min walk distance, *%LAA* percent low attenuation area at −950 Hounsfield units, *BODE* BMI, airflow obstruction, dyspnea and exercise capacity index, *FACIT* functional assessment of chronic illness therapy fatigue score

### C4Ma3 may be superior to fibrinogen in predicting mortality

**Fig. 3:**
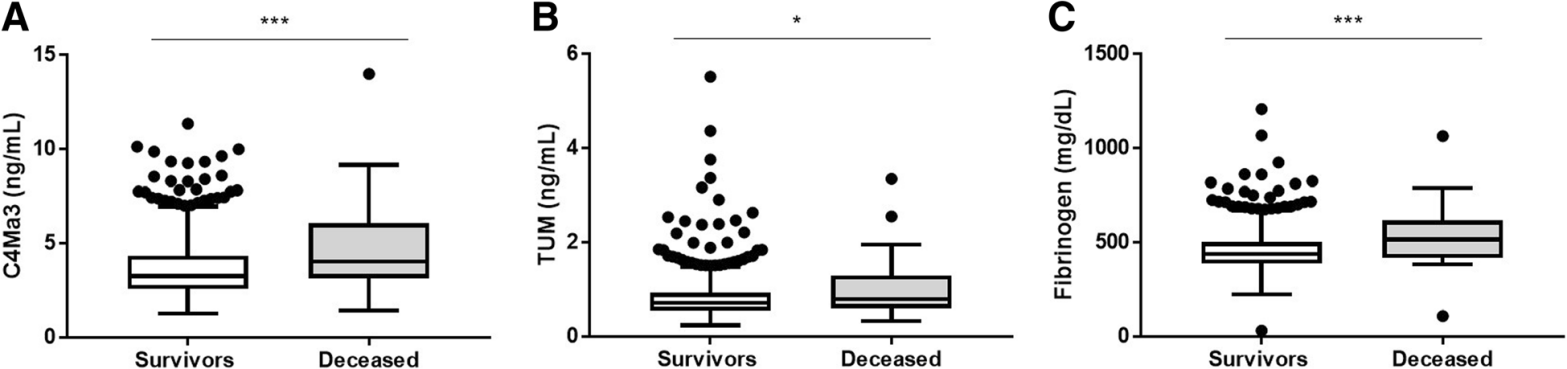
Plasma C4Ma3 (**a**), plasma TUM (**b**) and plasma fibrinogen (**c**) levels in survivors (*n* = 954) versus deceased (*n* = 30) COPD subjects. Data are shown as Tukey box-and-whiskers plot with a line indicating median, the box indicating the 25th and 75th percentiles (the interquartile range, IQR), and whiskers indicating 1.5 times the IQR. Data points outside this limit are shown by individual points. Asterisks indicate statistical significance: ***p < 0.001

**Fig. 4:**
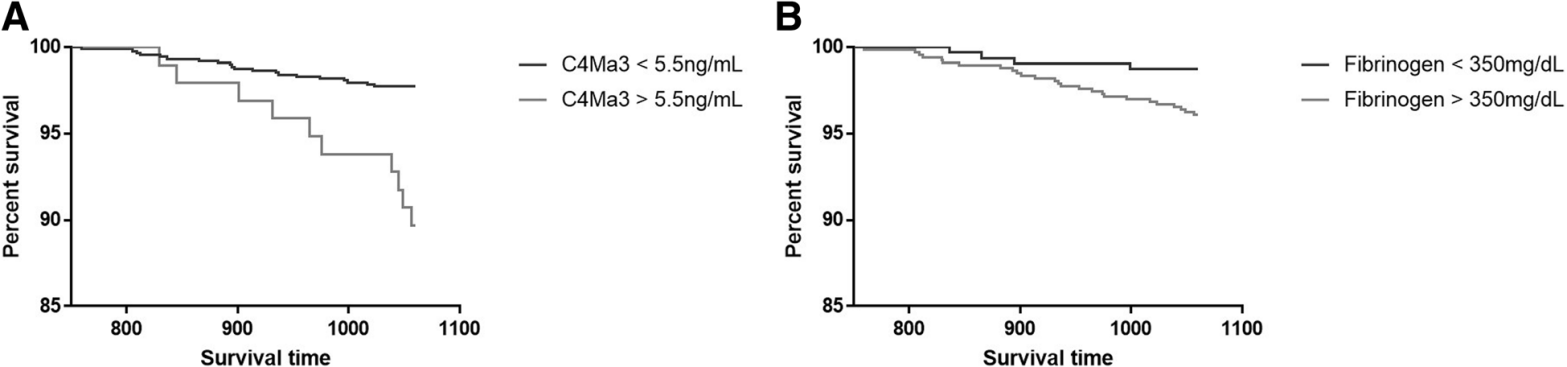
Kaplan-Meier survival curve analysis using the cut-off 5.5 ng/mL plasma C4Ma3 (**a**) and the cut-off of 350 mg/dL plasma fibrinogen (**b**)

COPD subjects which were registered as dead as compared to alive at the end of the study period had significantly higher levels of plasma C4Ma3, TUM and fibrinogen (*P* = 0.0008, *P* = 0.0467 and *P* = 0.0006, respectively) (Table 2/Fig. 3). Univariate Cox proportional hazard regression showed age, smoking status, 6-min walk distance (6MWD), modified Medical Research Council (mMRC) dyspnea score, and prior hospitalizations due to acute exacerbations to influence mortality risk (Table 3). Cox regression analysis adjusted for age, smoking status, prior hospitalizations due to exacerbations, mMRC and 6MWD showed that plasma C4Ma3 was an independent predictor of mortality with a hazard ratio of 1.57 (95% CI 1.29–1.92) per SD increase (*P* < 0.0001), whereas TUM showed not to be an independent predictor of mortality. Plasma fibrinogen showed similar results as C4Ma3 with a hazard ratio of 1.62 (95% CI 1.31–1.99) per SD increase (*P* < 0.0001) (Table 3). Plasma C4Ma3 values were dichotomized into a high and a low group based on a ROC curve, which was analyzed by Kaplan-Meier survival curve (Fig. 4a). For comparison, plasma fibrinogen was analyzed using the published cut-off value of 350 mg/dL (Fig. 4b) [18]. Cox proportional hazards regression was used for assessing the risk of mortality in high vs low group in a crude and adjusted analysis. The hazard ratio for the high plasma C4Ma3 group was 4.68 (95% CI 1.40–15.71, *P* < 0.0001). Here, 10.3% of subjects in the high group died while only 2.3% of the subjects died in the low C4Ma3 group. The hazard ratio for the high plasma fibrinogen group was 3.30 (95% CI 1.55–7.03, *P* = 0.0186). In the adjusted analysis high C4Ma3 level, age, and former smoking were included in the model while prior hospitalized acute exacerbations, mMRC dyspnea score and 6MWD were not significant. This model provided a hazard ratio for being in the high plasma C4Ma3 group of 5.12 (95% CI 2.28–11.50, *P* = 0.0001). A similar model with high plasma fibrinogen level showed fibrinogen to be non-significant and instead, the model included age, former smoking and mMRC dyspnea score. In addition, adding both high C4Ma3 and high Fibrinogen to a model only included age, smoking and plasma C4Ma3 providing a hazard ratio for being high in plasma C4Ma3 of 3.75 (95% CI 1.60–8.80, *P* = 0.0024). High levels of fibrinogen showed to be non-significant (Table 4**).**

**Table 3:** Cox regression in relation to mortality risk

Parameter	Hazard ratio (95% CI)	*P* value
Age	1.14 (1.0654–1.2201)	0.0001
BMI	1.01 (0.9548–1.0752)	0.6640
BODE	1.17 (0.9828–1.3958)	0.0774
Current smoking	0.17 (0.0564–0.6132)	0.0057
6MWD	0.99 (0.99–1.00)	0.0205
mMRC dyspnea score	1.55 (1.11–2.17)	0.0106
Prior AECOPD	1.00 (0.74–1.37)	0.98
Prior hospitalizations due to AECOPD	1.52 (1.02–2.26)	0.0375
C4Ma3 per SD	1.65 (1.33–2.03)	< 0.0001
C4Ma3 per SD (adjusted for confounders)	1.57 (1.29–1.92)	< 0.0001
TUM per SD	1.307 (1.08–1.58)	0.0064
Fibrinogen per SD	1.58 (1.28–1.94)	< 0.0001
Fibrinogen per SD (adjusted for confounders)	1.62 (1.31–1.99)	< 0.0001

Cox regression in relation to mortality risk. *BMI* body mass index, *BODE* BMI, airflow obstruction, dyspnea and exercise capacity index, *6MWD* 6-min walk distance, *mMRC* modified Medical Research Council score, *AECOPD* acute exacerbations of chronic pulmonary disease. Confounders included age, current smoking status, 6MWD, mMRC score and prior hospitalizations due to AECOPD

**Table 4:** Cox regression using cut-off levels for C4Ma3 and fibrinogen

Parameter	Hazard ratio (95% CI)	*P* value
Model 1 (high C4Ma3)		
High C4Ma3	5.12 (2.28–11.50)	0.0001
Age	1.11 (1.03–1.19)	0.0051
Former smoking	5.54 (1.30–23.60)	0.0206
Model 2 (high Fibrinogen)		
Age	1.10 (1.03–1.19)	0.0079
Former smoking	4.82 (1.12–20.65)	0.0342
mMRC dyspnea score	1.45 (1.03–2.04)	0.0334
Model 3 (high C4Ma3 and high Fibrinogen)		
High C4Ma3	3.75 (1.60–8.80)	0.0024
Age	1.09 (1.02–1.17)	0.0145
Former smoking	5.03 (1.17–21.62)	0.0301

Cox proportional hazards regression, enter method. Parameters tested for inclusion in all the models: High biomarker level; Age; smoking status; prior hospitalized acute exacerbations; *mMRC* modified Medical Research Council dyspnea score and *6MWD* 6-min walk distance

### Combining C4Ma3 and fibrinogen improves prediction of mortality

To investigate if the added effects of plasma C4Ma3 and fibrinogen could provide additional predictive value, we forced the two biomarkers into a model in addition to age and smoking status. Cox proportional hazards regression was used to assess the risk of mortality in high vs low groups in an adjusted analysis defined by a ROC curve. This model provided a hazard ratio for being in the high plasma C4Ma3*Fibrinogen group of 5.74 (95% CI 2.65–12.41), *P* < 0.0001, which was higher than both plasma C4Ma3 and plasma fibrinogen individually. Age and former smoking were included in the model while prior hospitalized acute exacerbations, mMRC dyspnea score and 6MWD were not. (Table 5).

**Table 5:** Cox regression combining C4Ma3 and fibrinogen

Parameter	Hazard ratio (95% CI)	*P* value
Model 4 (High C4Ma3*Fibrinogen)		
High C4Ma3*Fibrinogen	5.74 (2.65–12.41)	< 0.0001
Age	1.11 (1.03–1.19)	0.0072
Former smoker	5.37 (1.26–22.86)	0.0231

Cox proportional hazards regression, enter method. Parameters tested for inclusion in all the models: High biomarker level; Age; smoking status; prior hospitalized acute exacerbations; *mMRC* modified Medical Research Council score and *6MWD* 6-min walk distance

### C4Ma3 is related to hospitalized exacerbations

**Fig. 5:**
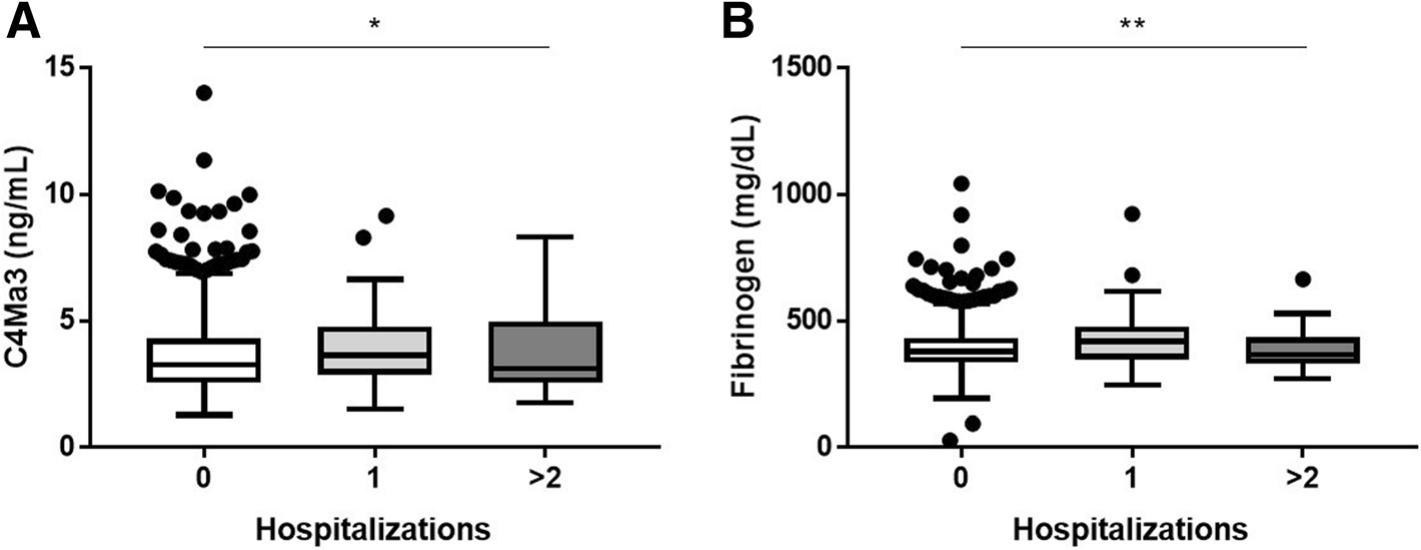
Plasma C4Ma3 (**a**) and plasma fibrinogen (**b**) levels in COPD subjects with no hospitalizations (*n* = 839), one hospitalization (*n* = 81) and more than 2 hospitalizations (*n* = 28) in the year prior to study start. Data are shown as Tukey box-and-whiskers plot with a line indicating median, the box indicating the IQR) and whiskers indicating 1.5 times the IQR. Asterisks indicate statistical significance: *p < 0.05, **p < 0.01, ***p < 0.001 and ****p < 0.0001

Plasma C4Ma3 levels were related to the number of hospitalizations due to COPD exacerbations in the year prior to study start (Kruskal-Wallis *P* = 0.0375) **(**Fig. 5a). Levels were not able to predict any future self-reported exacerbations (data not shown). Plasma fibrinogen levels were related to hospitalized exacerbations prior to study start (Kruskal-Wallis *P* = 0.0058) **(**Fig. 5b**)** as well as being related to future exacerbations in the year following biomarker measurement (Kruskal-Wallis *P* ≥ 0.0001) (data not shown). To find out if high plasma C4Ma3 and high plasma fibrinogen alone were able to predict exacerbations in the following year, a logistic regression was performed. BMI, airflow Obstruction, Dyspnea and Exercise capacity index (BODE) and mMRC dyspnea score were included in the model while age, former smoking, and 6MWD were not significant. Only the BODE index was able to predict exacerbations in the following year. This was also the case when including high C4Ma3*Fibrinogen in the model.

## Discussion

Identifying patients at highest risk of progression or the highest disease activity and determining responders versus non-responders to a disease-modifying drug in expensive clinical trials would be valuable to COPD subjects. Biomarkers of disease activity are not only needed to accelerate drug development but also to understand the heterogeneity of COPD, which can make it difficult to diagnose and choose the right treatment for each patient. We benchmarked the only FDA approved blood-based biomarker of COPD, plasma fibrinogen, with biomarkers reflecting BM remodeling by measuring the degradation of COL4A3, by plasma C4Ma3 and TUM. We found that C4Ma3 was highly associated with all-cause mortality and showed to be superior to fibrinogen in predicting mortality since COPD subjects being high in C4Ma3 had a poorer prognosis than those with low C4Ma3 levels. More interestingly, combining C4Ma3 and fibrinogen was able to improve the prediction of mortality compared to C4Ma3 and fibrinogen alone. In contrast, plasma fibrinogen was related to future self-reported exacerbations whereas C4Ma3 was only related to prior hospitalized exacerbations. The combination of fibrinogen and C4Ma3 was not able to predict future exacerbations either. Furthermore, the data indicated the importance of COL4A3 fragments, since the results for C4Ma3 and TUM were different.

The rate of BM remodeling is proposed to be increased during periods of high disease activity and faster disease progression [19]. Furthermore, it has been shown by Schumann et al. that plasma C4Ma3 levels were increased in COPD subjects from the stable state to the exacerbation and in a hospitalized exacerbation compared to a moderate exacerbation [20]. We showed in this study that plasma C4Ma3 levels were related to previous hospitalized acute exacerbations, which may indicate that plasma C4Ma3 may represent continuous disease activity that results from previous severe events. Plasma fibrinogen was related to hospitalized exacerbations but also self-reported exacerbations, indicating that fibrinogen is more related to exacerbations than C4Ma3. Furthermore, fibrinogen was related to future exacerbations, which was not the case for C4Ma3.

Disease activity is not necessarily correlated with disease severity, patients with severe airflow limitation may not decrease further in FEV_1_ and thereby not be high in disease activity [21, 22]. Since C4Ma3 was proposed as a disease activity marker no correlation was observed with levels of airflow limitation as defined by FEV_1_. Fibrinogen showed similar results. Instead, this study showed that plasma C4Ma3 was an independent predictor of mortality outcome. Using a cut-off level of 5.5 ng/mL for C4Ma3, dividing the COPD subjects into high versus low biomarker levels, showed that COPD subjects with high biomarker levels had a poorer prognosis than those with low biomarker levels. Fibrinogen was not able to predict mortality when using the already published cut-off value of 350 mg/dL in a larger cohort [18] and this may reflect a weaker predictive value.

Fibrinogen is an acute phase soluble plasma glycoprotein, synthesized primarily in the liver and proteolytically cleaved by thrombin into fibrin during blood coagulation, making it a potential biomarker for systemic chronic wound healing [23]. COL4A3 is abundantly expressed in the normal alveolar and glomerular BM, but not in the rest of the body, making C4Ma3 and TUM more specific to the lung. Moreover, COL4A3 has been shown to inhibit the activation of neutrophils and endothelial cell proliferation, and to induce cell apoptosis by its NC1 domain, tumstatin [24–26]. Tumstatin has been shown to have antiangiogenic effects by blocking the interaction between vascular endothelial growth factor (VEGF) and α_V_β_3_ integrin leading to apoptosis of proliferating endothelial cells [27, 28]. This suggests that COL4A3 may be involved in the development of COPD by modulating the inflammatory response or alveolar cell apoptosis [24]***.*** This supports the idea that measuring COL4A3 degradation is interesting for COPD not only by predicting mortality and hospitalized exacerbations but also with a potential role in understanding pathways associated with the pathogenesis of COPD. Notably, the data highlight the importance of knowing the specific fragment that are measured, since the results for C4Ma3 and TUM were different.

Fibrinogen and the biomarker C4Ma3 reflect two different aspects of COPD and by combining the two markers COPD patients with chronic wound healing and remodeling in the basement membrane of the lungs may be identified. We, therefore, speculated that combining the two markers would be a superior prognostic predictor relative to fibrinogen and C4Ma3 alone. C4Ma3*Fibrinogen improved the prediction of mortality but did not add to the predictive value for self-reported exacerbations.

A key limitation of our study is that data were generated for a subpopulation of the full ECLIPSE study comprising the study participants that progress the least and the most in terms of FEV_1_ decline over 3 years which may explain the low number of mortality events and reduced statistical power. Despite this, we found significant associations with mortality for C4Ma3 which seems promising and worthy of replication in larger studies. Additionally, baseline samples were not available, shortening the true follow-up time to two years. However, as this was an observational study it should not interfere with the interpretation of the results. Here, we compared a cutoff for C4Ma3 found by ROC curve in the current study with a published cutoff for fibrinogen. To determine if the associations are applicable to the general COPD population and to confirm if C4Ma3 is superior to fibrinogen in predicting mortality, the results should, therefore, be confirmed in an independent cohort.

In conclusion, we were able to compare fibrinogen, C4Ma3 and TUM as biomarkers of disease activity in COPD, alone and in combination. We showed that high levels of plasma C4Ma3 were associated with poorer prognosis and that plasma C4Ma3 levels were related to hospitalization due to exacerbation prior to study start. Plasma fibrinogen was not related to mortality using the published cut-off value of 350 mg/dL but showed to be associated with both hospitalized and self-reported exacerbations and future exacerbations. Furthermore, combining C4Ma3 and fibrinogen improved the prediction of mortality, but the combination was not related to future exacerbations. These results indicate that C4Ma3 may be able to identify COPD subjects with high disease activity and poor prognosis alone or in combination with the current FDA approved biomarker plasma fibrinogen.

## Additional file

Additional file 1: **Table S1.** Ethics and review boards. (PDF 169 kb)

## Abbreviations

6MWD: 6-min walk distance
AECOPD: Acute exacerbations of chronic pulmonary disease
AUC: Area under the curve
BM: Basement membrane
BMI: Body mass index
BODE: Body-mass-index, airflow obstruction, dyspnea and exercise capacity index
C4Ma3: Biomarker of MMP-mediated degradation of the type IV collagen alpha 3 chain
COL4A3: The alpha 3 chain of type IV collagen
COPD: Chronic obstructive pulmonary disease
ECLIPSE: Evaluation of COPD longitudinally to identify predictive surrogate end-points
ECM: Extracellular matrix
FDA: U.S. Food and Drug Administration
FEV_1_: Forced expiratory volume in the first second
FVC: Forced vital capacity
GOLD: Global initiative for chronic obstructive lung disease
IPF: Idiopathic pulmonary fibrosis
LLA: Low areas of attenuation below 950 Hounsfield unit
MMP: Matrix metalloproteinases
mMRC: Modified Medical Research Council dyspnea scale
NC1: C-terminal non-collagenous domain of type IV collagen
ROC: Receiver operating curve
SD: Standard deviation
SGRQ: St. George’s respiratory questionnaire
TUM: Tumstatin
VEGF: Vascular endothelial growth factor
