# Supplementary material for: Type IV collagen turnover is predictive of mortality in COPD: a comparison to fibrinogen in a prospective analysis of the ECLIPSE cohort
Source: Respir Res. 2019 Apr 1;20:63. doi: 10.1186/s12931-019-1026-x (PMC6444812; doi:10.1186/s12931-019-1026-x)
Supplement: Supplementary file 1 — Table S1. Ethics and review boards. (PDF 169 kb) [file 12931_2019_1026_MOESM1_ESM.pdf]

Type IV collagen turnover is predictive of mortality in COPD: A comparison to fibrinogen in a prospective analysis of the ECLIPSE cohort

Jannie Marie Bülow Sand[1*], Sarah Rank Rønnow[1,2*#], Lasse Løcke Langholm[1,3], Tina Manon-Jensen[1], Morten Asser Karsdal[1], Ruth Tal-Singer[4£], Bruce E. Miller[4£], Jørgen Vestbo[5£], Diana Julie Leeming[1]

[£]The Evaluation of COPD Longitudinally to Identify Surrogate Endpoints (ECLIPSE) study, investigators.
***Equal contributors**
[#]Corresponding author

[1]Nordic Bioscience A/S, Herlev, Denmark, [2]University of Southern Denmark, The Faculty of Health Science, Odense, Denmark, [3]University of Copenhagen, the faculty of health and medical sciences, [4]GSK R&D, Collegeville, PA, USA, [5]Division of Infection Immunity and Respiratory Medicine, The University of Manchester, Manchester Academic Health Science Centre, and Manchester University NHS Foundation Trust, Manchester, England.

Supplementary

Table 1. Ethics and review boards

Inv/Site No.	Institution & Address	IEC/IRB Committee
027904/023622	Asthma Centre<br>Ivan Vazov Street Nr 31<br>PO Box 1018<br>Pleven, 5800 Bulgaria	Ethics Committee for Multicentre Trials<br>8, Damyan Gruev str.,<br>Sofia 1303<br>Bulgaria
076209/023623	Military Medical Academy<br>Georgi Sofiiski 3 str<br>Sofia 1606 Bulgaria	Ethics Committee for Multicentre Trials<br>8, Damyan Gruev str.,<br>Sofia 1303<br>Bulgaria
006269/023794	Montreal Chest Institute<br>3650 St-Urbain, Room K307<br>Montreal, QC H2X 2P4 Canada	McGill University Health Center<br>Research Ethics Board<br>3650 St Urbain<br>Montreal, QC H2X 2P4
006610/023668	The Lung Center<br>2775 Laurel St., 7th Floor<br>Vancouver, BC V5Z 1M9 Canada	The University of British Columbia<br>Office of Research Services Clinical Research Ethics Board<br>Room210, 828 West 10th Ave<br>Vancouver, BC V5Z 1L8 Canada
004970/023483	Queen Elizabeth II Health Sciences Centre<br>Halifax Infirmary<br>1796 Summer St., Room 5452<br>Halifax, NS B3H 3A7 Canada	Capital Health Research Ethics Board<br>Centre for Clinical Research Building<br>118-5790 University Avenue<br>Halifax, NS B3H 1V7 Canada
029347/023796	McMaster University, Health Sciences Center<br>1200 Main St. West, Room 3U25<br>Hamilton, ON L8N 3Z5 Canada	Hamilton Health Sciences/Faculty of Health Sciences Research Ethics Board<br>293 Wellington St N,<br>Suite 102<br>Hamilton, Ontario L8L 8E7<br>Canada
035031/023795	Pacific Lung Health Center<br>1081 Burrard Street<br>8B Providence Wing<br>Vancouver, BC V6Z 1Y6 Canada	Office of Research Services (ORS)<br>Providence Health Care Research Institute<br>Room 1125, 11th Floor<br>1190 Hornby Street c/o 1081 Burrard Street<br>Vancouver, BC V6Z 1Y6
006193/023547	Hopital Laval, Recherche Clinic<br>Centre de Pneumologie<br>2725 Chemin Sainte Foy<br>Pavillion U, Locale U 1751<br>Sainte Foy, QC G1V 4G5 Canada	Comité d'éthique de la recherche<br>Institut Universitaire de Cardiologie et de Pneumologie de Québec (IUCPQ)<br>2725, chemin Ste-Foy<br>Quebec, Qc<br>Canada<br>G1V 4G5
004395/02409 4	Kingston General Hospital<br>Richardson House<br>102 Stuart Street<br>Kingtson, ON K7L 2V6 Canada	Queens University<br>Office of Research Services<br>Fleming Hall, Jemmett Wing, Room 301<br>Queens University<br>Kingston, ON, Canada
000309/024204	SPLiN s.r.o.<br>Oddeleni TRN<br>Cimicka 37/446<br>Praha 8 18200 Czech Republic	Multicentric Ethics Committee Fakultni nemocnice v Motole<br>V Uvalu 84 Prague 5 ZIP: 150 06<br>Czech Republic
000683/023960	H:S Hvidovre Hospital<br>Hjerte-Lungemedicinsk afdeling<br>Kettegaard Alle 30<br>Opgang 1<br>Hvidovre 2650 Denmark	Den videnskabsetiske komité for region hovedstaden<br>Regionsgaarden<br>Kongensvænge 2<br>3400 Hillerød
001687/024403	Astmacentrum Hornerheide<br>Hornerheide 1<br>Horn 6085 NM Netherlands	METC Zuidwest-Holland<br>M.H.H.A. Kirkels-Breukers<br>P.O Box 5011<br>2600 GA Delft<br>The Netherlands

082272/023579	Haukeland Universitets sykehus<br>Chest department<br>Jonas Liesvei 65<br>Bergen N 5021 Norway	Regional Ethic Committee West<br>Haukeland University Hospital, N-5021 Bergen, Norway
014566/024144	P3 Research Bown Hospital<br>Churchill Drive Crofton Downs<br>Wellington 6035 New Zealand	c/- Ministry of Health<br>1-3 The Terrace<br>Level 1<br>Wellington<br>6011
136098/024146	KOPA Golnik<br>Golnik 36<br>4204 Golnik Slovenia	The National Medical Ethics Committee of the Republic of Slovenia<br>University Institute of Clinical Neurophysiology, Medical Center Ljubljana,<br>Zaloška c. 7,<br>SI-1525 Ljubljana
108244/026658	Hospital Son Dureta<br>C/ Andrea Doria 55<br>Palma de Mallorca 07014 Spain	Comité ètic d´investigació clínica Illes Balears<br>Conselleria de Salut i Consum<br>Direcció General d'Avaluació i Acreditació<br>Comitè Ètic d'Investigació Clínica de les Illes Balears (CEIC-IB)<br>Camí de Jesús, 38 A<br>07011 Palma - Illes Balears
000473/023973	Aintree University Hospitals NHS Foundation Trust<br>Respiratory Research Department<br>Longmoor Lane, Ward 14a<br>Liverpool L9 7AL United Kingdom	Oxfordshire REC C<br>2nd Floor, Astral House<br>Chaucer Business Park<br>Granville Way<br>Bicester OX26 4JT
082424/023706	Cambridge Institute for Medical Research<br>Department of Medicine<br>Hills Road, Wellcome Trust / MRC Building<br>Cambridge CB2 2XY United Kingdom	Oxfordshire REC C<br>2nd Floor, Astral House<br>Chaucer Business Park<br>Granville Way<br>Bicester OX26 4JT
029855/023707	New Royal Infirmary of Edinburgh<br>Little France Crescent, Old Dalkeith Road<br>51 Little France Crescent<br>Edinburgh Midlothian EH16 4SA<br>United Kingdom	Oxfordshire REC C<br>2nd Floor, Astral House<br>Chaucer Business Park<br>Granville Way<br>Bicester OX26 4JT
023731/023974	Wythenshawe Hospital<br>Medicine Evaluation Unit<br>Southmoor Road<br>The Langley Building, North West Lung Research Centre<br>Manchester M23 9LT United Kingdom	Oxfordshire REC C<br>2nd Floor, Astral House<br>Chaucer Business Park<br>Granville Way<br>Bicester OX26 4JT
029742/024037	The Royal Free Hospital<br>Academic Unit of Respiratory Medicine<br>Pond Street<br>London NW3 2QG United Kingdom	Oxfordshire REC C<br>2nd Floor, Astral House<br>Chaucer Business Park<br>Granville Way<br>Bicester OX26 4JT
001069/024393	Institute of Phthisiatry and Pulmonology<br>Department of Pulmonology<br>10, Amosova Str<br>Kiev 03680 Ukraine	
001047/024392	Institute of Phthisiatry and Pulmonology<br>Department of Pulmonology<br>10, Amosova Str<br>Kiev 03680 Ukraine	
001071/024362	Institute of Phthisiatry and Pulmonology<br>Department of Pulmonology<br>10, Amosova Str<br>Kiev 03680 Ukraine	
001063/024364	Donetsk State Medical University<br>Department of Therapy<br>16 Illicha prospect<br>Donetsk 83003 Ukraine	

044783/023140	University of Texas Health Science Center<br>Pulmonary Diseases<br>7400 Merton Minter Blvd., (111E)<br>San Antonio, TX 78229 United States	University of Texas Health Science Center<br>7703 Floyd Curl Drive, Mail Code 7830<br>San Antonio, TX 78229-3900
077534/023146	Rhode Island Hospital<br>Division of Pulmonary, Sleep & Critical Care Medicine<br>593 Eddy Street, APC 7th Floor<br>Providence, RI 02903 United States	Lifespan Office of Research Administration<br>167 Point Street<br>Providence, RI 02903
013075/023147	Los Angeles Biomedical Research Institute at Harbor-UCLA Medical Center<br>Rehab Clinical Trials Center<br>1124 W. Carson St., Bldg. J4<br>Torrance, CA 90502 United States	John F. Wolf, MD Human Subjects Committee<br>Los Angeles Biomedical Research Institute at Harbor-UCLA Medical Center<br>1124 West Carson Street<br>Torrance, CA 90502
008578/023354.	St. Elizabeth's Medical Center<br>Pulmonary STN-3<br>736 Cambridge Street<br>Boston, MA 02135 Unites States	Research/Human Subjects Committee<br>Caritas St. Elizabeth's Medical Center<br>Cambridge St., HOQ3<br>Boston, MA 02135
021992/023148	Pulmonary Associates of Richmond, Inc.<br>1000 Boulders Parkway, Suite 201<br>Richmond, VA 23225 United States	Goodwyn Institution Review Board<br>9380 Main Street<br>Cincinnati, OH 45242
010875/023149	Pulmonary Associates, PA<br>1112 East McDowell Road<br>Phoenix, AZ 85006 United States	Goodwyn Institution Review Board<br>9380 Main Street<br>Cincinnati, OH 45242
011553/023150	Advances in Medicine<br>42362 Bob Hope Drive<br>Rancho Mirage, CA 92270 Unites States	Western International Review Board<br>3535 Seventh Ave SW<br>Olympia, WA 98508
010094/023355	Baylor Clinic-Baylor College of Medicine<br>6620 Main Street<br>Suite 11B, 16<br>Houston, TX 77030 United States	Baylor College of Medicine IRB<br>Clinical Research Studies<br>One Baylor Plaza, Mail stop 600D<br>Houston, TX 77030
015497/023356	Dartmouth-Hitchcock Medical Center<br>Pulmonary & Critical Care Center<br>One Medical Center Drive<br>Lebanon, NH 03756 Unites States	Dartmouth-Hitchcock Medical Center<br>Committee for the Protection of Human Subjects<br>11 Rope Ferry Road #6210<br>Hanover, NH 03755
008005/023357	National Jewish Medical & Research Center<br>Weinberg Clinical Research Unit<br>1400 Jackson Street<br>Denver, CO 80206 United States	National Jewish Medical & Research Center IRB<br>1400 Jackson Street<br>Denver, CO 80206
009021/023358	University of Nebraska Medical Center<br>Pulmonary Clinical Studies Unit<br>982465 Nebraska Medical Center<br>DRC II1022<br>Omaha, NE 68198 United States	University of Nebraska Medical Center IRB<br>Academic & Research Services Bldg. 3000<br>987830 Nebraska Medical Center<br>Omaha, NE 68198
083482/023571	Yale University School of Medicine<br>Internal Medicine/Pulmonary<br>1 Gilbert Street, TAC S 441<br>New Haven, CT 06520 United States	Yale University School of Medicine<br>Human Investigation Committee<br>47 College Street, Suite 204<br>New Haven, CT 06520
015449/023359	Mayo Clinic<br>Pulmonary Clinical Research Center<br>Lanmark 2-46<br>14 – 2nd Street SW<br>Rochester, MN 55905 United States	Mayo Foundation IRB<br>201 Building, Room 4-60<br>200 First Street SW<br>Rochester, MN 55905
080801/023389	Creighton University Medical Center<br>Pulmonary & Critical Care Division<br>601 N. 30th Street, Suite 3820<br>Omaha, NE 68131 United States	Creighton University Medical Center IRB<br>2500 California Plaza<br>Omaha, NE 68178

010532/023489	University of Pittsburgh Medical Center<br>Emphysema Research Center<br>3471 5th Ave., Suite 1211<br>Pittsburgh, PA 15213 United States	University of Pittsburgh IRB<br>3500 Fifth Ave, Ground Level<br>Pittsburgh, PA 15213

021093/023390	Houston VA Medical Center<br>2002 Holcombe Blvd.<br>Pulmonary 111-1, Room 3C-220<br>Houston, TX 77030 United States	Baylor College of Medicine IRB<br>Clinical Research Studies<br>One Baylor Plaza, Mail stop 600D<br>Houston, TX 77030
008864/023392	Midwest Chest Consultants, PC<br>330 First Capital Drive, Suite 470<br>St. Charles, MO 63301 United States	Goodwyn Institution Review Board<br>9380 Main Street<br>Cincinnati, OH 45242
077533/023391	Harvard University-Brigham & Women's Hospital<br>Channing Laboratory<br>181 Longwood Ave.<br>Boston, MA 02115 United States	Brigham & Women's Hospital IRB<br>Partners Human Research Office<br>116 Huntington Ave, Suite 1002<br>Boston, MA 02116
016586/023393	University of Miami School of Medicine<br>1600 NW 10th Ave, #7064-A (R-47)<br>Miami, FL 33136	Western International Review Board<br>3535 Seventh Ave SW<br>Olympia, WA 98508
057745/023394	Johns Hopkins Asthma & Allergy Center<br>5501 Hopkins Bayview Circle, Room 3B-58<br>Baltimore, MD 21224 United States	John Hopkins School of Medicine<br>Office of Human Subject Research<br>1620 McElderry St., Reed Hall<br>Suite B 130<br>Baltimore, MD 21205
012252/023395	St. Francis Hospital & Medical Center<br>Pulmonary Medicine<br>114 Woodland Street<br>Hartford, CT 06105 United States	St. Francis Hospital & Medical Center IRB<br>Department of Research<br>114 Woodland St.<br>Hartford, CT 06105
